# Draft Genome Sequence of the Marine-Derived, Anticancer Compound-Producing Bacterium *Streptomyces* sp. Strain GMY01

**DOI:** 10.1128/mra.01366-20

**Published:** 2023-05-04

**Authors:** Jaka Widada, Ema Damayanti, Camelia Herdini, Nastiti Wijayanti, Akira Hosoyama, Atsushi Yamazoe, Chiho Suzuki-Minakuchi, Bambang Hariwiyanto, Sofia Mubarika, Achmad Dinoto, Mustofa, Hideaki Nojiri

**Affiliations:** a Department of Agricultural Microbiology, Faculty of Agriculture, Universitas Gadjah Mada, Yogyakarta, Indonesia; b Research Center for Food Technology and Processing, National Research and Innovation Agency, Yogyakarta, Indonesia; c Faculty of Medicine, Public Health and Nursing, Universitas Gadjah Mada, Yogyakarta, Indonesia; d Faculty of Biology, Universitas Gadjah Mada, Yogyakarta, Indonesia; e Biological Resource Center, National Institute of Technology and Evaluation, Nishihara, Shibuya-ku, Tokyo, Japan; f Research Center for Applied Microbiology, National Research and Innovation Agency, Cibinong, Indonesia; g Biotechnology Research Center, The University of Tokyo, Bunkyo-ku, Tokyo, Japan; h Collaborative Research Institute for Innovative Microbiology, The University of Tokyo, Tokyo, Japan; Montana State University

## Abstract

The marine *Streptomyces* sp. strain GMY01 was isolated from Indonesian marine sediment. Genome mining analysis revealed that GMY01 has 28 biosynthetic gene clusters, dominated by genes encoding nonribosomal peptide synthetase and polyketide synthase.

## ANNOUNCEMENT

Marine actinobacteria are biotechnologically valuable organisms sourced from the ocean (1). Various marine actinobacteria, especially those belonging to the genus *Streptomyces*, have been isolated from marine sediments and sponge species (2). In the past 10 years, the discovery of new compounds from marine *Streptomyces* spp. has led to the further development of novel antibacterial, antimicrobial, and anticancer compounds (3–7).

The novel *Streptomyces* strain GMY01 was isolated from a marine sediment sample from Krakal Beach (Yogyakarta, Indonesia; 8°8′44″S, 110°35′59″E) (5). Strain GMY01 has high anticancer activity in lung cancer cells (A549) (6). In this study, we present the annotated genome sequence of the bacterium GMY01 and report the potential active compounds encoded by its biosynthetic gene clusters. GMY01 was isolated using solid starch nitrate medium, which was prepared using filtered seawater and contained 75 μg/mL cycloheximide (5). The GMY01 genome was obtained from cells grown in NBRC 227 medium on plates at 28°C until fully grown, and DNA was obtained using the Qiagen DNA tissue kit and EZ1 Advanced instrument (7). The genomic DNA of strain GMY01 was sequenced using a combined shotgun sequencing method with the 454 GS FLX Titanium system (Roche) and paired-end (PE) sequencing using the HiSeq 1000 platform (Illumina), and DNA was obtained as previously reported (8). Default parameters were used for all software unless otherwise noted. Read lengths of 500 bp (454) and 100 bp (Illumina; paired-end format) were used for library preparation, with the 454 GS FLX Titanium library preparation kit and TruSeq DNA PCR-free HT library prep kit, respectively. The sequences were assembled using Newbler v2.6, and assembled sequences with a low quality value (Q < 40) were visually checked and removed using Consed v25. Using Newbler v2.6 and Consed v25, 270,000 Roche 454 reads (135 Mb; length, 500 bp) and 500,000 Illumina reads (900 Mb; length, 200 bp) were obtained. Ten initial scaffolds were obtained using Newbler v2.6 (Roche; overlapMinMatchIdentity = 98; allContigThresh = 0), with 1 million PE reads (234 Mb) and 0.54 million mate pair (MP) reads (98 Mb) for Illumina and Roche 454, respectively, that were quality filtered using ShortReadManager v0.995 (threshold values, read score = 30; read length = 21; 21-mer frequency = 2) (9).

Full-genome sequencing of strain GMY01 led to an assembly of 73 contigs, for a total genome size of 7.97 Mb and a predicted GC content of 73.22%. Annotation was performed using RAST (10, 11) and the NCBI Prokaryotic Genome Annotation Pipeline (PGAP) v4.11 (best-placed reference protein set; GeneMarkS-2Z+; https://www.ncbi.nlm.nih.gov/genome/annotation_prok/) and identified a total of 6,774 genes, including 6,697 coding DNA sequences (CDSs), 77 RNAs, 68 tRNAs, and 3 noncoding RNAs (ncRNAs).

The average nucleotide identity based on a BLAST+ comparison (ANIb) was calculated using the JSpecies software tool (12). The ANIb results showed that *Streptomyces* sp. GMY01 had an ANIb value of 82.22% with Streptomyces leeuwenhoekii (formerly *Streptomyces* sp. strain C34) (GenBank accession number LN831790.1), a novel actinomycete isolated from a saline area (13). Genome mining analysis using antiSMASH v6.0 (14) revealed that GMY01 has 28 biosynthetic gene clusters (BGCs). These BGCs were predicted to be involved in the production of flaviolin, geosmin, ectoine, class IV lanthipeptide/SflA, albaflavenone, and informatipeptin, with 100% deduced amino acid sequence similarity.

### Data availability.

This whole-genome shotgun project has been deposited at DDBJ/ENA/GenBank under accession number JABBNA000000000 and BioSample accession number SAMN14667424. The version described in this paper is JABBNA010000000. The raw sequence reads are available under BioProject accession number PRJNA625871.
